# Deep learning methods in protein structure prediction

**DOI:** 10.1016/j.csbj.2019.12.011

**Published:** 2020-01-22

**Authors:** Mirko Torrisi, Gianluca Pollastri, Quan Le

**Affiliations:** a Centre for Applied Data Analytics Research, University College Dublin, Ireland; b School of Computer Science, University College Dublin, Ireland

**Keywords:** Deep learning, Protein structure prediction, Machine learning

## Abstract

Protein Structure Prediction is a central topic in Structural Bioinformatics. Since the ’60s statistical methods, followed by increasingly complex Machine Learning and recently Deep Learning methods, have been employed to predict protein structural information at various levels of detail. In this review, we briefly introduce the problem of protein structure prediction and essential elements of Deep Learning (such as Convolutional Neural Networks, Recurrent Neural Networks and basic feed-forward Neural Networks they are founded on), after which we discuss the evolution of predictive methods for one-dimensional and two-dimensional Protein Structure Annotations, from the simple statistical methods of the early days, to the computationally intensive highly-sophisticated Deep Learning algorithms of the last decade. In the process, we review the growth of the databases these algorithms are based on, and how this has impacted our ability to leverage knowledge about evolution and co-evolution to achieve improved predictions. We conclude this review outlining the current role of Deep Learning techniques within the wider pipelines to predict protein structures and trying to anticipate what challenges and opportunities may arise next.

## 1 Introduction

Proteins hold a unique position in Structural Bioinformatics. In fact, the origins of the field itself can be traced to Max Perutz and John Kendrew’s pioneering work to determine the structure of globular proteins (which also led to the 1962 Nobel Prize in Chemistry) [1], [2]. The ultimate goal of Structural Bioinformatics, when it comes to proteins, is to unearth the relationship between the residues forming a protein and its function, i.e., in essence, the relationship between genotype and phenotype. The ability to disentangle this relationship can potentially be used to identify, or even design, proteins able to bind specific targets [3], catalyse novel reactions [4] or guide advances in biology, biotechnology and medicine [5], e.g. editing specific locations of the genome with CRISPR-Cas9 [6].

According to Anfinsen’s thermodynamic hypothesis, all the information that governs how proteins fold is contained in their respective primary sequences, i.e. the chains of amino acids (AA, also called residues) forming the proteins [7], [8]. Anfinsen’s hypothesis led to the development of computer simulations to score protein conformations, and, thus, search through potential states looking for that with the lowest free energy, i.e. the native state [9], [8]. The key issue with this energy-driven approach is the explosion of the conformational search space size as a function of a protein’s chain length. A solution to this problem consists in the exploitation of simpler, typically coarser, abstractions to gradually guide the search, as proteins appear to fold locally and non-locally at the same time but incrementally forming more complex shapes [10].

**Fig. 1:**
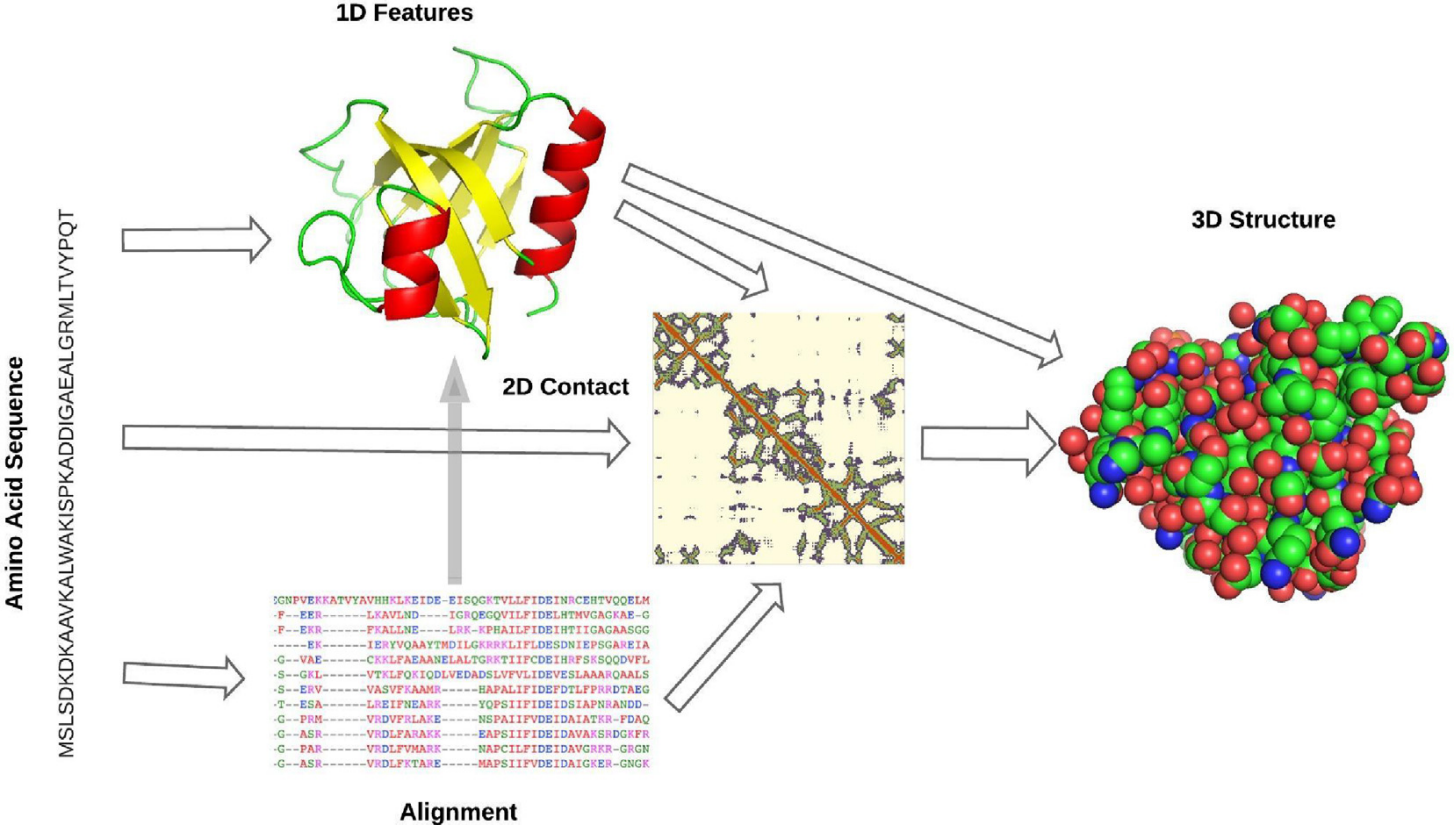
A generic pipeline for ab initio Protein Structure Prediction, in which evolutionary information in the form of alignments, 1D and 2D PSA are intermediate steps.

A standard pipeline for Protein Structure Prediction envisages intermediate prediction steps where abstractions are inferred which are simpler than the full, detailed 3D structure, yet structurally informative - what we call Protein Structure Annotations (PSA) [11]. The most commonly adopted PSA are secondary structure, solvent accessibility and contact maps. The former two are one-dimensional (1D) abstractions which describe the arrangement of the protein backbone, while the latter is a two-dimensional (2D) projection of the protein tertiary structure in which any 2 AA in a protein are labelled by their spatial distance, quantised in some way (e.g. greater or smaller than a given distance threshold). Several other PSA, e.g. torsion angles or contact density, and variations of the aforementioned ones, e.g. half-sphere exposure and distance maps, have been developed to describe protein structures [11]. Fig. 1 depicts a pipeline for the prediction of protein structure from the sequence in which the intermediate role of 1D and 2D PSA is highlighted.

It should be noted that protein intrinsic disorder [12], [13], [14] can be regarded as a further 1D PSA with an important structural and functional role [15], which has been predicted by Machine Learning and increasingly Deep Learning methods similar to those adopted for the prediction of other 1D PSA properties [16], [17], [18], [19], [20], [21], [22], sometimes alongside them [23]. However, given its role in protein structure prediction pipelines is less clear than for other PSA, we will not explicitly focus on disorder in this article and refer the reader to specialised reviews on disorder prediction, e.g. [24], [25], [26].

**Fig. 2:**
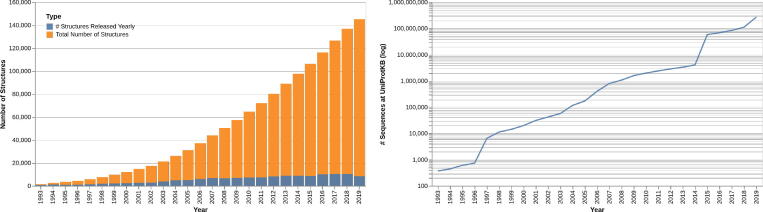
Growth of known structures in the Protein Data Bank (left) and known sequences in Uniprot (right). The y-axis is shown in logarithmic scale for the Uniprot.

The slow but steady growth in the number of protein structures available at atomic resolution has led to the development of PSA predictors relying also on homology detection (“template-based predictors”), i.e. predictors directly exploiting proteins of known structure (“templates”) that are considered to be structurally similar based on sequence identity [27], [28], [29], [30]. However, a majority PSA predictors are “ab initio”, that is, they do not rely on templates. Ab-initio predictors leverage extensive evolutionary information searches at the sequence level, relying on ever-growing data banks of known sequences and constantly improving algorithms to detect similarity among them [31], [32], [33]. Fig. 2 shows the growth in the number of known structures in the Protein Data Bank (PDB) [34] and sequences in the Uniprot [35] - the difference in pace is evident, with an almost constant number of new structures having been added to the PDB each year for the last few years while the number of known sequences is growing close to exponentially.

### 1.1 Feed forward neural networks

A Feed Forward Neural Network (FFNN) is an artificial neural network [36] containing no cycles. In particular layered FFNN are FFNN whose nodes can be partitioned into groups (layers) that are ordered and in which the outputs of layer *i* are inputs to and only to layer i+1. The first layer is known as Input layer, the last as Output layer and any layer in between is a Hidden layer whose units form an intermediate representation of an instance. Layered FFNN, which may be trained from examples using the back-propagation algorithm [36] and have been proven to have universal approximation properties [37], have been used to predict 1D PSA since the ’80s [38], [39], [40]. These networks have typically been used in their so-called “windowed” version, in which each segment of a fixed number of amino acids in a sequence is treated as the input for a separate example, the target for the segment being the PSA of interest for one of the amino acids in the segment (usually the central one).

### 1.2 Deep Learning

Deep Learning [41] is a sub-field of Machine Learning based on artificial neural networks, which emphasises the use of multiple connected layers to transform inputs into features amenable to predict corresponding outputs. Given a sufficiently large dataset of input–output pairs, a training algorithm can be used to automatically learn the mapping from inputs to outputs by tuning a set of parameters at each layer in the network.

While in many cases the elementary building blocks of a Deep Learning system are FFNN or similar elementary cells, these are combined into deep stacks using various patterns of connectivity. This architectural flexibility allows Deep Learning models to be customised for any particular type of data. Deep Learning models can generally be trained on examples by back-propagation [36], which leads to efficient internal representations of the data being learned for a task. This automatic feature learning largely removes the need to do manual feature engineering, a laborious and potentially error-prone process which involves expert domain knowledge and is required in other Machine Learning approaches. However, Deep Learning models easily contain large numbers of internal parameters and are thus data-greedy - the most successful applications of Deep Learning to date have been in fields in which very large numbers of examples are available [41]. In the remainder of this section we summarise the main Deep Learning modules which are used in previous research in Protein Structure Prediction.

Convolutional Neural Networks (CNN) [42] are an architecture designed to process data which is organised with regular spatial dependency (like the tokens in a sequence or the pixels in an image). A CNN layer takes advantage of this regularity by applying the same set of local convolutional filters across positions in the data, thus brings two advantages: it avoids the overfitting problem by having a very small number of weights to tune with respect to the input layer and the next layer dimensionality, and it is translation invariant. A CNN module is usually composed of multiple consecutive CNN layers so that the nodes at later layers have larger receptive fields and can encode more complex features. It should be noted that “windowed” FFNN discussed above can be regarded as a particular, shallow, version of CNN, although we will keep referring to them as FFNN in this review to follow the historical naming practice in the literature.

Recurrent Neural Networks (RNN) [43] are designed to learn global features from sequential data. When processing an input sequence, a RNN module uses an internal state vector to summarise the information from the processed elements of the sequence: it has a parameterised sub-module which takes as inputs the previous internal state vector and the current input element of the sequence to produce the current internal state vector; the final state vector will summarise the whole input sequence. Since the same function is applied repeatedly across the elements of a sequence, RNN modules easily suffer from the gradient vanishing or gradient explosion problem [44] when applying the back propagation algorithm to train them. Gated recurrent neural network modules like Long Short Term Memory (LSTM) [45] or Gated Recurrent Unit (GRU) [46] are designed to alleviate these problems. Bidirectional versions of RNNs (BRNN) are also possible [47] and particularly appropriate in PSA predictions, where data instances are not sequences in time but in space and propagation of contextual information in both directions is desirable.

Even though the depth of a Deep Learning model increases its expressiveness, increasing depth also makes it more difficult to optimise the network weights due to gradients vanishing or exploding. In [48] Residual Networks have been proposed to solve these problems. By adding a skip connection from one layer to the next one, a Residual Network is initialised to be near the identity function thus avoids large multiplicative interactions in the gradient flow. Moreover, skip connections act as “shortcuts”, providing shorter input–output paths for the gradient to flow in otherwise deep networks.

## 2 Methods for 1D Protein Structural Annotations

First generation PSA predictors relied on statistical calculations of propensities of single AA towards structural conformations, usually secondary structures [49], [50], [51], [52], which were then combined into actual predictions via hand-crafted rules. While these methods predicted at better than chance accuracy, they were quite limited - especially on novel protein structures [53], with per-AA accuracies usually not exceeding 60%.

In a second generation of predictors [54], information from more than one AA at a time was fed to various methods, including FFNN to predict secondary structure [38], [39], and least squares, i.e. a standard regression analysis, to predict hydrophobicity values [55]. This step change was made possible by the increasing number of resolved structures available. These methods were somewhat more accurate than first generation ones, with secondary structure accuracies of 63–64% reported [38].

The third generation of PSA predictors has been characterised by the adoption of evolutionary information [56] in the form of alignments of multiple homologous sequences as input to the predictive systems, which are almost universally Machine Learning, or Deep Learning algorithms. One of the early systems from this generation, PHD [56], arguably the first to predict secondary structure at over 70% accuracy, was implemented as two cascaded FFNN taking segments of 13 AA and 17 secondary structure predictions as inputs, containing 5,000–15,000 free tunable parameters, and trained by back-propagation.

Subsequent sources of improvement were more sensitive tools for mining evolutionary information such as PSI-BLAST [32] or HMMER [57], and the ever increasing nature of both the databases of available structures and sequences, with PSIPRED [58], based on a similar stack of FFNN to that used in PHD, albeit somewhat larger, achieving state of the art performances at the time of development, with sustained 76% secondary structure prediction accuracy.

### 2.1 Deep Learning methods for 1D PSA prediction

Various Deep Learning algorithms have been routinely adopted for PSA prediction since the advent of the third generation of predictors [11], alongside more classic Machine Learning methods such as k-Nearest Neighbors [63], [64], Linear Regression [65], Hidden Markov Models [66], Support Vector Machines (SVM) [67] and Support Vector Regression [68].

PHD, PSIPRED, and JPred [69] are among the first notable examples in which cascaded FFNN are used to predict 1D PSA, in particular secondary structure. DESTRUCT [70] expands on this approach by simultaneously predicting secondary structure and torsion angles by an initial FFNN, then having a filtering FFNN map first stage predictions into new predictions, and then iterating, with all copies of the filtering network sharing their internal parameters.

SPIDER2 [59] builds on this approach adding solvent accessibility to the set of features predicted and training an independent set of weights for each iteration. The entire set of PSA predicted is used, along with the input features of the first stage, to feed the second and third stage. Each stage is composed of a window-based (w  = 17) 3-layered FFNN with 150 hidden units each [59].

SSpro is a secondary structure predictor based on a Bidirectional RNN architecture followed by a 1D CNN stage. The architecture was shown to be able to identify the terminus of the protein sequence and was quite compact with only between 1400 and 2900 free parameters [47]. Subsequent versions of SSpro increased the size of the training datasets and networks [71]. Similar architectures have been implemented to predict solvent accessibility and contact density [72]. The latest version of SSpro adds a final refinement step based on a PSI-BLAST search of structurally similar proteins [30], i.e. is a template-based predictor.

A variant to plain BRNN-CNN architectures are stacks of Recurrent and Convolutional Neural Networks [73], [27], [74], [31], [75]. In these a first BRNN-CNN stage is followed by a second structurally similar stage fed with averages over segments of predictions from the first stage. Porter, PaleAle, BrownAle and Porter+ (Brewery) are Deep Learning methods employing these architectures to predict secondary structure, solvent accessibility, contact density and torsion angles, respectively [60], [11]. The latest version of Porter (v5) is composed by an ensemble of 7 models with 40,000–60,000 free parameters each, using multiple methods to mine evolutionary information [31], [76]. The same architecture has also been trained on a combination of sequence and structural data [27], [28], and in a cascaded approach similar to that of DESTRUCT and SPIDER2 in which multiple PSA are predicted at once and the prediction is iterated [77].

SPIDER3 [61] substitutes the FFNN architecture of SPIDER2 with a Bidirectional RNN with LSTM cells [45] followed by a FFNN, predicts 4 PSA at once, and iterates the prediction 4 times. Each of the 4 iterations of SPIDER3 is made of 256 LSTM cells per direction per layer, followed by 1024 and 512 hidden units per layer in the FFNN. Adam optimiser and Dropout (with a ratio of 50%) [78] are used to train the over 1 million free parameters of the model. SPIDER2 and SPIDER3 are the only described methods which employ seven representative physio-chemical properties in input along with both HHblits and PSI-BLAST outputs.

### 2.2 Convolutional neural networks

RaptorX-Property is a collection of 1D PSA predictors released since 2010 and based on Conditional Neural Fields (CNF), i.e. Neural Networks possessing an output layer made of Conditional Random Fields (CRF) [79]. The most recent version of RaptorX-Property is based on Deep Convolutional Neural Fields (DeepCNF), i.e. CNN with CRF output [80], [23]. This version has 5 convolutional layers containing 100 hidden units with a window size of 11 each, i.e. roughly 500,000 free parameters (10 times and 100 times as many as Porter5 and PHD, respectively). The latest version of RaptorX-Property depends on HHblits instead of PSI-BLAST for the evolutionary information fed to DeepCNF models [23].

NetSurfP-2.0 is a recently developed predictor which employs either HHblits or MMsEqs. 2 [76], [81], depending on the number of sequences in input [62]. NetSurfP-2.0 is made of two CNN layers, consisting of 32 filters with 129 and 257 units, respectively, and two BRNN layers, consisting of 1024 LSTM cells per direction per layer. The CNN input is fed to the BRNN stage as well. NetSurfP-2.0 predicts secondary structure, solvent accessibility, torsion angles and structural disorder with a different fully connected layer per PSA.

**Fig. 3:**
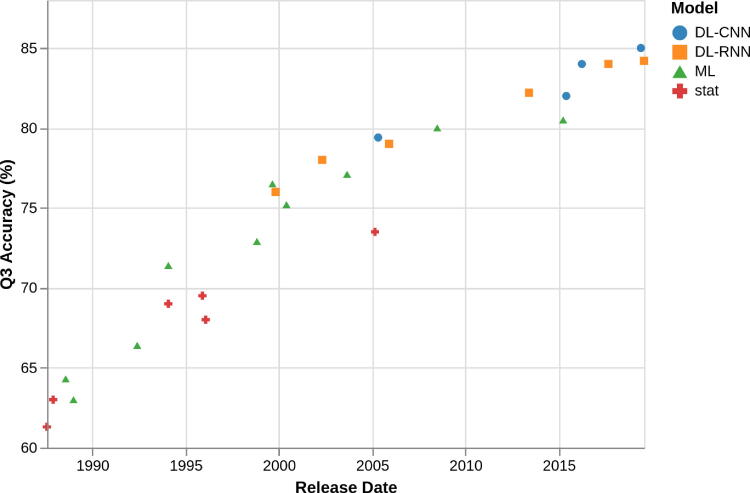
Performances of secondary structure predictors over the years. “stat” are predictors based on statistical methods other than Neural Networks. “ML” are predictors based on shallow Neural Networks or Support Vector Machines. “DL-CNN” are Deep Learning methods based on Convolutional Neural Networks. “DL-RNN” are Deep Learning methods based on Recurrent Neural Networks. Data extracted from accompanying publications of predictors referenced in this article.

In Fig. 3 we report a scatterplot of performances of secondary structure predictors vs. the year of their release. Gradual, continuing improvements are evident from the plot, as well as the transition from statistical methods to classical Machine Learning and later Deep Learning methods. A set of surveys of recent methods for the prediction of protein secondary structure can be found in [82], [83], [84], [85] and a thorough comparative assessment of high-throughput predictors in [86].

## 3 Methods for 2D Protein Structural Annotations

A typical pipeline to predict protein structure envisages a step in which 2D PSA of some nature are predicted [11]. In fact, most of the recent progress in Protein Structure Prediction has been driven by Deep Learning methods applied to the prediction of contact or distance maps [87], [88].

Contact maps have been adopted to reconstruct the full three-dimensional (3D) protein structure since the ’90s [89], [90], [91]. Although the 2D-3D reconstruction is known to be a NP-hard problem [92], heuristic methods have been devised to solve it approximately [89], [93], [94] and optimised for computational efficiency [90]. The robustness of these heuristic methods has been tested against noise in the contact map [95].

Distance maps and multi-class contact maps (i.e. maps in which distances are quantised into more than 2 states) typically lead to more accurate 3D structures than binary maps and tend to be more robust when random noise is introduced in the map [29], [96]. Nonetheless, one contact every twelve residues may be sufficient to allow robust and accurate topology-level protein structure modeling [97].

Predicted contact maps can also be helpful to score and, thus, guide the search for 3D models [98].

One of the earliest examples of 2D PSA annotations are β-sheet pairings, i.e. AA partners in parallel and anti-parallel β-sheet conformations. Machine/Deep Learning methods such as FFNN [99], BRNN [100] and multi-stage approaches [101] have been used since the late ’90s to predict whether any 2 residues are partners in a β-sheet. Similarly, disulphide bridges (formed by cysteine-cysteine residues) have been predicted by the Edmonds-Gabow algorithm and Monte Carlo simulation annealing [102], or hybrid solutions such as Hidden Markov Models and FFNN [103], and multi-stage FFNN, SVM and BRNN [104], alongside classic Machine Learning models such as SVM [105], pure Deep Learning models such as BRNN [106], and FFNN [107].

The prediction of a contact map’s principal eigenvector (using BRNN) is instead an example of 1D PSA used to infer 2D characteristics [108]. The predictions of β-sheet pairings, disulphide bridges and principal eigenvectors have been prompted by the need for “easy-to-predict”, informative abstractions which can be used to guide the prediction of more complex 2D PSA such as contact or distance maps. Ultimately, however, most interest in 2D PSA has been in the direct prediction of contact and distance maps as these contain most, if not all, the information necessary for the reconstruction of a protein’s tertiary structure [89], [29], [96], while being translation and rotation invariant [91] which is a desirable property for the target of Machine Learning and Deep Learning algorithms.

Early methods for contact map prediction typically focused on simple, binary maps, and relied on statistical features extracted from evolutionary information in the form of alignments of multiple sequences. Features such as correlated mutations, sequence conservation, alignment stability and family size were inferred from multiple alignments and were shown to be informative for contact map prediction since the ’90s [109], [110]. Early methods often relied on simple linear combinations of features, though FFNN [111] and other Machine Learning algorithms such as Self-Organizing Maps [112] and SVM [113] quickly followed.

### 3.1 Modern and deep learning methods for 2D PSA prediction

2D-BRNN [72], [124] are an extension to the BRNN architecture used to predict 1D PSA. These models, which are designed to process 2D maps of variable sizes, have 4 state vectors summarising information about the 4 cardinal corners of a map. 2D-BRNN have been applied to predict contact maps [72], [124], [108], [125], multi-class contact maps [29], and distance maps [96]. Contact map predictions by 2D-BRNN have also been refined using cascaded FFNN [126]. Both ab initio and template-based predictors have been developed to predict maps (as well as 1D PSA) [29], [96]. In particular, template-based contact and distance map predictors rely both on the sequence and structural information and, thus, are often better than ab initio predictors even when only dubious templates are available [29], [96].

More recently, growing abundance of evolutionary information data and computational resources has led to substantial breakthroughs in contact map prediction [127]. More sophisticated statistical methods have been developed to calculate mutual information without the influence of entropy and phylogeny [128], co-evolution coupling [129], direct-coupling analysis (DCA) [130] and sparse inverse covariance estimation [131]. The ever-growing number of known sequences has led to the development of more optimised and, thus, faster tools [132] able to also run on GPU [133]. PSICOV [131], FreeContact [132] and CCMpred [133], which are notable results of this development, have allowed the exploitation of ever growing data banks and prompted a new wave of Deep Learning methods.

MetaPSICOV is a notable example of a Deep Learning method applied to PSICOV, FreeContact and CCMpred, as well as 1D features (such as predicted 1D PSA) [134]. MetaPSICOV is a two-stage FFNN with one hidden layer with 55 units. MetaPSICOV2, the following version, is a two-stage FFNN with two hidden layers with 160 units each and also a template-based predictor [114].

DeepCDpred is a multi-class contact map ab initio predictor which attempts to extend MetaPSICOV [115]. In particular, PSICOV is substituted with QUIC - a similarly accurate but significantly faster implementation of the sparse inverse covariance estimation - and the two-stage FFNN with an ensemble of 7 deeper FFNN (with 8 hidden layers) which are trained on different targets and, thus, result in a multi-class map predictor.

RaptorX-Contact is one of the first examples of contact map predictor based on a Residual CNN architecture [116]. RaptorX-Contact has been trained on CCMpred, mutual information, pairwise potential extraction and RaptorX-Property’s output, i.e. secondary structure and solvent accessibility predictions [23]. RaptorX-Contact uses filters of size 3×3 and 5×5, 60 hidden units per layer and a total of 60 convolutional layers.

DNCON2 is a two-stage CNN trained on a set of input features similar to MetaPSICOV [117]. The first stage is composed of an ensemble of 5 CNN trained on 5 different thresholds, which feeds a following refining stage of CNN. The first stage of DNCON2 can be seen as a multi-class contact map predictor.

DeepContact (also known as i_Fold1) aims to demonstrate the superiority of CNN over FFNN to predict contact maps [118]. DeepContact is a 9-layer Residual CNN with 32 filters of size 5×5 trained on the same set of features used by MetaPSICOV. The outputs of the third, sixth and ninth layers are concatenated with the original input and fed to a last hidden layer to perform the final prediction.

DeepCov uses CNN to predict contact maps when limited evolutionary information is available [119]. In particular, DeepCov has been trained on a very limited set of input features: pair frequencies and covariance. This is one of the first notable examples of 2D PSA predictors which entirely skips the prediction of 1D PSA in its pipeline.

PconsC4 is a CNN with limited input features to significantly speed-up prediction time [120]. In particular, PconsC4 uses predicted 1D PSA, the GaussDCA score, APC-corrected mutual information, normalised APC-corrected mutual information and cross-entropy. PconsC4 requires only a recent version of Python and a GCC compiler with no need for any further external programs and appears to be significantly faster (and more accurate) than MetaPSICOV [120], [114].

SPOT-Contact has been inspired by RaptorX-Contact and extends it by adding a 2D-RNN stage downstream of a CNN stage [121]. SPOT-Contact is an ensemble of models based on 120 convolutional filters – half 3×3 and half 5×5 – followed by a 2D-BRNN with 800 units – 200 LSTM cells for each of the 4 directions – and a final hidden layer composed of 400 units. Adam, a 50% dropout rate and layer normalization are among the Deep Learning techniques implemented to train this predictor. CCMpred, mutual and direct-coupling information are used as inputs as well as the output of SPIDER3, i.e. predictions of solvent accessibility, half-Sphere exposures, torsion angles and secondary structure [61].

TripletRes [122] is a contact map predictor that ranked first in the Contact Predictions category of the latest edition of CASP, a bi-annual blind competition for Protein Structure Prediction [135]. TripletRes is composed of 4 CNN trained end-to-end. More in detail, 3 coevolutionary inputs, i.e. the covariance matrix, precision matrix and coupling parameters of the Potts model, are fed to 3 different CNN which are then fused in a unique CNN downstream. Each CNN is composed of 24 residual convolutional layers with a kernel of size 3×3×64. The training of TripletRes required 4 GPUs running concurrently - using Adam and a 80% dropout rate. TripletRes successfully identified and predicted both globally and locally multi-domain proteins following a divide et impera strategy.

AlphaFold [123] is a Protein Structure Prediction method that achieved the best performance in the Ab initio category of CASP13 [135]. Central to AlphaFold is a distance map predictor implemented as a very deep residual neural networks with 220 residual blocks processing a representation of dimensionality 64×64×128 – corresponding to input features calculated from two 64 amino acid fragments. Each residual block has three layers including a 3×3 dilated convolutional layer – the blocks cycle through dilation of values 1, 2, 4, and 8. In total the model has 21 millions parameters. The network uses a combination of 1D and 2D inputs, including evolutionary profiles from different sources and co-evolution features. Alongside a distance map in the form of a very finely-grained histogram of distances, AlphaFold predicts Φ and Ψ angles for each residue which are used to create the initial predicted 3D structure. The AlphaFold authors concluded that the depth of the model, its large crop size, the large training set of roughly 29,000 proteins, modern Deep Learning techniques, and the richness of information from the predicted histogram of distances helped AlphaFold achieve a high contact map prediction precision.

**Fig. 4:**
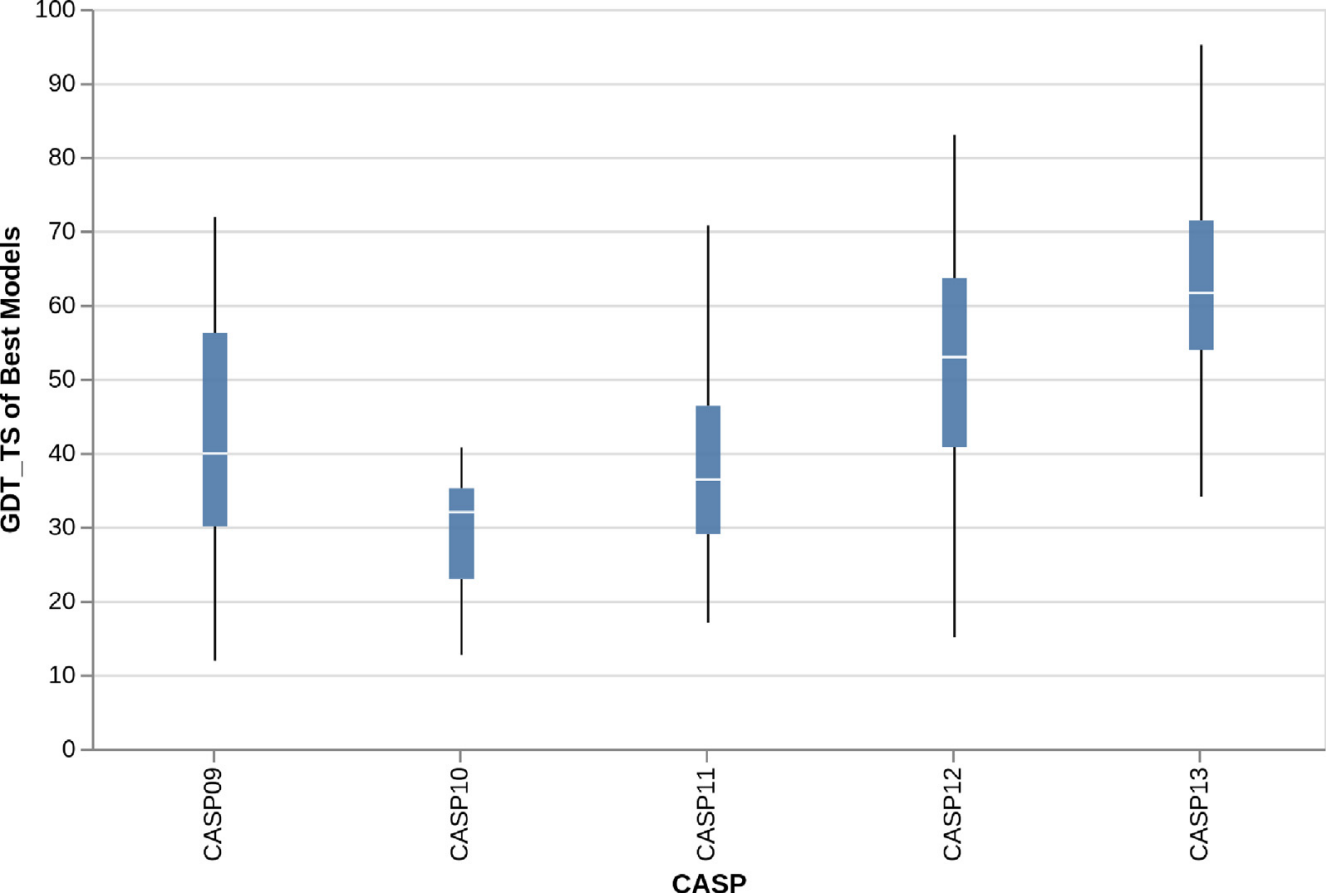
Improvements in quality of 3D predictions for free modelling (ab initio) targets between CASP9 and CASP13.

Constant improvements in contact and distance map predictions over the last few years have directly resulted in improved 3D predictions. Fig. 4 reports the average quality of predictions submitted to the CASP competition for free modelling targets, i.e. proteins for which no suitable templates are available and predictions are therefore fully ab initio, between CASP9 (2010) and CASP13 (2018). Improvements especially over the last two editions are largely to be attributed to improved map predictions [127], [136].

## 4 Summary and outlook

Proteins fold spontaneously in 3D conformations based only on the information present in their residues [7]. Protein Structure predictors are systems able to extract from the protein sequence information constraining the set of possible local and global conformations and use this to guide the folding of the protein itself. Deep Learning methods are successful at producing higher abstractions/representations while ignoring irrelevant variations of the input when sufficient amounts of data are provided to them [137]. Both characteristics together with the availability of rapidly growing protein databases increasingly make Deep Learning methods the preferred techniques to aid Protein Structure Prediction (see Tables 1 and 2). The highly complex landscape of protein conformations make Protein Structural Annotations one of the main research topics of interest within Protein Structure Prediction [11]. In particular, 1D annotations have been a central topic since the ’60s [1], [2] while the focus is progressively shifting towards more informative and complex 2D annotations such as contact maps and distance maps. This change of paradigm is mainly motivated by technological breakthroughs which result in continuous growth in computational power and protein sequences available thanks to next-generation sequencing and metagenomics [76], [81].

**Table 1:** Deep Learning methods for 1D PSA prediction, along with models adopted and tools to gather evolutionary information, respectively. Secondary structure (SS), solvent accessibility (SA), torsion angles (TA), contact density (CD) and disordered regions (DR) are the PSA predicted.

Predictor	PSA	Model	Evolutionary Information
SPIDER2 [59]	SS, SA	Multi-stage FFNN	PSI-BLAST
SSpro/ACCpro5 [30]	SS, SA	BRNN-CNN	PSI-BLAST
Brewery [60]	SS, SA, TA, CD	Multi-stage BRNN-CNN	PSI-BLAST, HHblits
SPIDER3 [61]	SS, SA, TA, CD	BLSTM	PSI-BLAST, HHblits
RaptorX-Property [23]	SS, SA, DR	CNF	PSI-BLAST, HHblits
NetSurfP-2.0 [62]	SS, SA, TA, DR	BLSTM	HHblits, (or) MMseqs2

**Table 2:** Modern and Deep Learning methods for 2D PSA prediction, along with models adopted and tools to gather evolutionary information, respectively. Contact maps (CM), multi-class CM and distance maps (DM) are the PSA predicted.

Predictor	PSA	Model	Evolutionary Information
MetaPSICOV2 [114]	CM	Multi-stage FFNN	HHblits, JackHMMer
DeepCDpred [115]	multi-class CM	Multi-stage FFNN	HHblits
RaptorX-Contact [116]	multi-class CM	Residual CNN	HHblits
DNCON2 [117]	CM	Multi-stage CNN	HHblits, jackHMMer
DeepContact [118]	CM	Residual CNN	HHblits, jackHMMer
DeepCov [119]	CM	CNN	HHblits
Pconsc4 [120]	CM	CNN	HHblits
SPOT-Contact [121]	CM	Residual CNN 2D-BLSTM	HHblits, PSI-BLAST
TripletRes [122]	CM	Multi-stage residual CNN	HHblits, jackHMMer, HMMER
AlphaFold [123]	DM	Residual CNN	HHblits, PSI-BLAST

Recent work on the prediction of 1D structural annotations [11], [31], [75], [61], contact map prediction [117], [122], and on overall structure prediction systems [123], [138], emphasises the importance of more sophisticated pipelines to find and exploit evolutionary information from ever growing databases. This is often achieved by running several tools to find multiple homologous sequences in parallel [32], [76], [81] and, increasingly, by deploying Machine/Deep Learning techniques to independently process the sequence before fusing their outputs into the final prediction. The correlation between sequence alignment quality and accuracy of PSA predictors has been empirically demonstrated [139], [140], [141]. How to best gather and process homologous sequences is an active research topic, e.g. RawMSA is a suite of predictors which proposes to substitute the pre-processing of sequence alignments with an embedding step in order to learn a representation of protein sequences instead of pre-compressing homologous sequences into input features [142].

The same trend towards end-to-end systems has been attempted in the pipeline from processed homologous sequences to 3D structure, e.g. in NEMO [143], a differentiable simulator, and RGN (Recurrent Geometrical Network) [144], an end-to-end differentiable learning of protein structure. However, state-of-the-art structure predictors are still typically composed of multiple intelligent systems. The last mile of Protein Structure Prediction, i.e. the building, ranking and scoring of structural models, is also fertile ground for Machine Learning and Deep Learning methods [145], [146]. E.g. MULTICOM exploits DNCON2 - a multi-class contact map predictor - to build structural models and to feed DeepRank - an ensemble of FFNN to rank such models [138]. DeepFragLib is, instead, a Deep Learning method to sample fragments (for ab initio structure prediction) [147]. The current need for multiple intelligent systems is supported by empirical results, especially in the case of hard predictions. Splitting proteins into composing domains, predicting 1D PSA, and optimising each component of the pipeline is particularly useful especially when alignment quality is poor [148].

Today, state-of-the-art systems for Protein Structure Prediction are composed by multiple specialised components [123], [138], [11] in which Deep Learning systems have an increasing, often crucial role, while end-to-end prediction systems entirely based on Deep Learning techniques, e.g. Deep Reinforcement Learning, may be on the horizon but are at present still immature. Progress in this field over the last few years has been substantial, even dramatic especially in the prediction of contact and distance maps [127], [136], but the essential role of structural, evolutionary, and co-evolutionary information in this progress cannot be understated, with ab initio prediction quality still lagging that of template-based predictions, proteins with poor alignments being still a weak spot and prediction of protein structure from a single sequence being a challenge that is far from solved [149], although some progress has recently been observed for proteins with shallow alignments [150]. More generally, given that our current structure prediction pipelines rely almost exclusively on increasingly sophisticated and sensitive techniques to detect similarity to known structures and sequences, it is unclear whether predictions truly represent low energy structures unless we know they are correct. The prediction of protein misfolding [151], [152] presents a further challenge for the current prediction paradigm, with Machine Learning methods only making slow inroads [153]. Nevertheless, as more computational resources, novel techniques and ultimately, critically, increasing amounts of experimental data will become available [137], further improvements are to be expected.

## Declaration of Competing Interest

The authors declare that they have no known competing financial interests or personal relationships that could have appeared to influence the work reported in this paper.
